# Polyphenol-Based Therapeutic Strategies for Mitochondrial Dysfunction in Aging

**DOI:** 10.3390/biom15081116

**Published:** 2025-08-03

**Authors:** Tamara Maksimović, Carmen Gădău, Gabriela Antal, Mihaela Čoban, Oana Eșanu, Elisabeta Atyim, Alexandra Mioc, Codruța Șoica

**Affiliations:** 1 Faculty of Pharmacy, “Victor Babes” University of Medicine and Pharmacy, 300041 Timisoara, Romania; tamara.maksimovic@umft.ro (T.M.); gabriela.antal@umft.ro (G.A.); mihaela.coban@umft.ro (M.Č.); oana.esanu@umft.ro (O.E.); atyimelisabeta@umft.ro (E.A.); alexandra.mioc@umft.ro (A.M.); codrutasoica@umft.ro (C.Ș.); 2 Research Center for Experimental Pharmacology and Drug Design (X-Pharm Design), “Victor Babes” University of Medicine and Pharmacy, 300041 Timișoara, Romania; 3 Department of General Surgery, Timiş County Emergency Clinical Hospital, 300723 Timisoara, Romania

**Keywords:** polyphenols, mitochondria, mitochondrial dysfunction, aging

## Abstract

Aging, a progressive and time-dependent decline in physiological functions, is driven by interconnected hallmarks, among which mitochondrial dysfunction plays a central role. Mitochondria not only regulate energy production but also play key roles in other cellular processes, including ROS generation, apoptosis, and metabolic signaling—all of which decline with aging. Polyphenols are a diverse group of natural compounds found in fruits, vegetables, tea, and wine; they emerged as promising anti-aging agents due to their ability to modulate several hallmarks of aging, particularly mitochondrial dysfunction. This review explores how various polyphenolic classes influence mitochondrial function and mitigate aging-related decline. These natural compounds have been shown to reduce oxidative stress, increase energy production, and help maintain normal mitochondrial structure. Moreover, in vitro and in vivo studies suggest that polyphenols can delay signs of aging and improve physical and cognitive functions. Overall, polyphenols show great potential to promote healthy aging and even delay the decline in physiological functions by protecting and enhancing mitochondrial health.

## 1. Introduction

**Figure 1:**
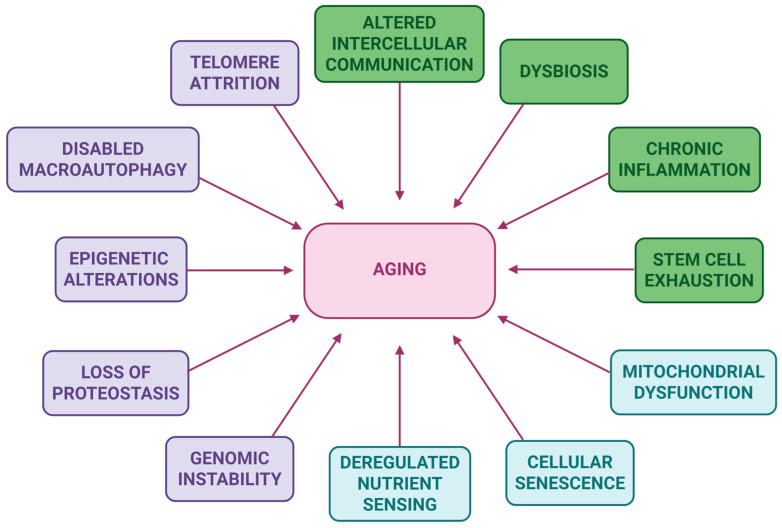
Aging: a complex process influenced by 12 interconnected hallmarks that cause time-dependent alterations, leading to aging. The aging hallmarks can be categorized in: (i) primary: genomic instability, telomere attrition, epigenetic alterations, loss of proteostasis, disabled macroautophagy (purple color), (ii) antagonistic: mitochondrial dysfunction, cellular senescence, deregulated nutrient sensing (blue color), and (iii) integrative: stem cell exhaustion, altered intercellular communication, chronic inflammation and dysbiosis (green color).

Aging is a complex, time-dependent decline of the physiological functions involved in homeostasis and overall biological integrity, driven by various mechanisms [1,2]. The aging process is marked by the loss of physiological functions, tissue deterioration, frailty, increased susceptibility to a number of pathologies, and, ultimately, increased vulnerability to death [3,4]. As life expectancy continuously increases, age-related pathologies—such as cancer and cardiovascular, neurodegenerative, and metabolic diseases—represent a serious challenge since they reduce life quality in elderly people [4,5]. Therefore, aging has come forth as an important research subject, as treating or reversing age-related alterations could potentially address all age-associated diseases simultaneously [6,7]. In this regard, one possible approach involves targeting the biological aging mechanisms, or hallmarks [8]; so far, 12 aging hallmarks have been identified, namely mitochondrial dysfunction, epigenetic alterations, genomic instability, telomere attrition, loss of proteostasis, cellular senescence, deregulated nutrient sensing, altered intercellular communication, stem cell exhaustion, chronic inflammation, disabled macroautophagy, and dysbiosis (Figure 1) [9].

As previously mentioned, mitochondrial dysfunction represents one of the aging hallmarks.Mitochondria are cellular organelles displaying two membranes, the outer mitochondrial membrane (OMM) and the inner mitochondrial membrane (IMM), which outline the intermembrane space. The inner mitochondrial membrane forms cristae, invaginations in the mitochondrial matrix that present four protein complexes and two electron carriers that constitute the electron transport chain (ETC) [10]. The mitochondrial matrix contains mitochondrial DNA (mtDNA), a circular, double-stranded molecule that encodes transfer RNA, ribosomal RNA, and respiratory complex subunits [11,12]. Mitochondria play a crucial role in a large number of cellular processes, including energy production, biomolecule synthesis, amino acid and fatty acid metabolism, cell signaling, apoptosis, and cell homeostasis [13,14].

As revealed by recent studies in the literature, during the aging process, mitochondria suffer numerous changes that lead to function impairment: increased reactive oxygen species (ROS) production, excessive mutations at the mtDNA level, loss of mitochondrial membrane potential, reduced ATP production, decreased enzymatic activity, changes in mitochondrial morphology, reduced mitochondrial number, impaired mitochondrial quality control, and accumulation of damaged mitochondria [4,15,16,17]. Therefore, targeting mitochondrial dysfunction represents one of the main strategies to slow down the aging process or even improve/treat the associated functional decline [18].

**Figure 2:**
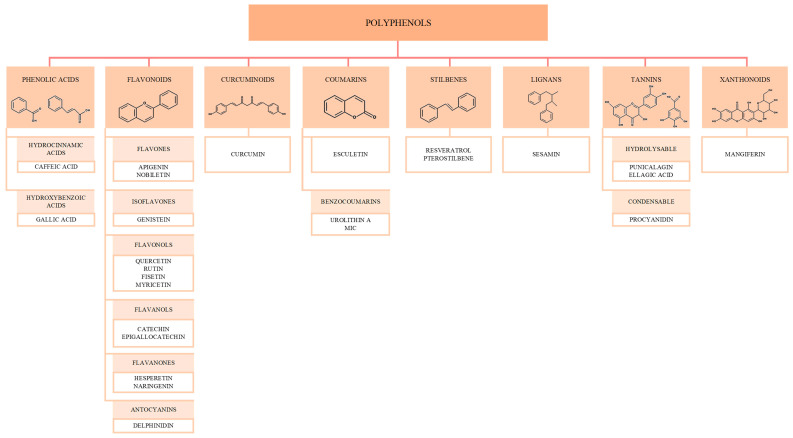
Polyphenols classification, based on the spatial arrangement of the phenolic rings and their substitutes. Phenolic acids consist of one ring, COOH and OH groups; flavonoids present two phenolic rings, connected by a C3 linker; coumarins consist of a benzopyrone scaffold; stilbenes, similarly to flavonoids, contain two phenolic rings, however, linked by a methylene group; lignans are formed from two benzylbutane moieties; tannins posses either galloyl esters or oligomeric/polymeric proanthocyanidins in their structure; xanthonoids contain two rings connected through oxygen and carbonyl group.

In the context of aging, polyphenols, a group of natural compounds, have emerged as potential anti-aging agents due to their ability to influence various aging hallmarks [19]. Polyphenols are secondary plant metabolites responsible for the color, aroma, and antioxidant effects of different vegetables and fruits [20] and are found in coffee, green tea, wine, apples, citrus fruits, berries, turmeric, beans, etc. [21]. Chemically, polyphenols contain at least two phenolic groups [20] and can be classified as phenolic acids, flavonoids, coumarins, stilbenes, lignans, or tannins (Figure 2) [22]. They can also be defined as compounds that derive from the shikimate/phenylpropanoid pathway and/or the polyketide pathway and lack nitrogen-based functions [20]. It has been shown that polyphenols have the ability to modulate a number of mitochondrial processes and pathways [23].

This paper aims to investigate the relationship between polyphenols, aging, and mitochondria while evaluating the therapeutic potential of polyphenols as anti-aging agents.

## 2. Polyphenols: Mechanisms and Anti-Aging Effects

There are several mechanisms that could explain the effect of polyphenols in the aging process: promotion of mitochondrial function, role in antioxidant signaling and NO bioavailability, prevention of cellular senescence, micro-RNA targeting, and modulation of autophagy and inflammation [19,24,25]. In fact, different subtypes of polyphenols can interact with different pathways that have roles in aging [26]. The influence of polyphenols on different aging hallmarks is presented in Table 1.

## 3. Mitochondria and Aging: Mechanisms Involved

### 3.1. ROS and Aging

Mitochondria are the site of ROS production [38]. ROS are a group of unstable free (superoxide anion O_2_^•−^, hydroxyl radical OH^•^) and non-free radicals (hydrogen peroxide, H_2_O_2_) mainly generated through oxidative phosphorylation [39]. More precisely, ROS are produced at the level of complexes I (CI) and III (CIII) of ETC as a consequence of electron leakage leading to the reduction of oxygen to superoxide [38]. In aged mitochondria, studies revealed that the electron leak increases due to several possible mechanisms, such as age-related activation of ROS generators (like xanthine oxidase, nitrogen oxide synthase); a shift in ROS nature, from low-reactivity oxygen species (hydrogen peroxide, superoxide) to high-reactivity oxygen species (hydroxyl radical); or production of ROS due to physiological signals [40]. While CI produces ROS at the flavin mononucleotide (FMN) site and N_2_ iron–sulfur cluster (redox centers localized on the matrix site of CI), CIII produces ROS on the both sides of the inner membrane [41,42].

**Figure 3:**
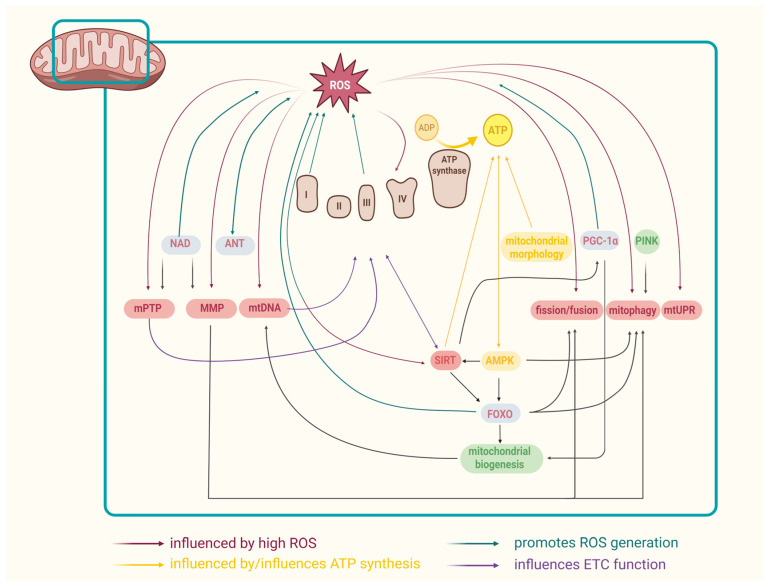
Mitochondrial mechanisms in aging and their interconnections: excessive ROS production (at the level of CI and CII) in aging leads to mPTP activation, MMP depolarisation, mtDNA mutations, reduced SIRT expression, ETS dysfunction, fission and fusion disturbance, mitophagy decline and mtUPR activation. On the other hand, reduced ANT activity, low NAD^+^ concentration, altered SIRT expression, decreased PGC-1α and FOXO levels promote ROS generation. In addition, SIRT influences the ROS, as well as ATP production, through ETC regulation. Hence, low SIRT concentration, along with changes in mitochondrial morphology and ETS dysfunction, causes a decline in ATP synthesis, which then activates AMPK in order to restore energy levels. Additionally, AMPK promotes mitophagy and activates SIRT and FOXO proteins. FOXO has the capability to influence mitophagy, fission and fusion. Moreover, many of the other mechanisms are interconnected: mPTP, when activated, inhibits oxidative phosphorylation; reduced MMP increases mitophagy and fission; mtDNA mutations decrease functionality of the respiratory chain complexes; deactivated PINK leads to a decline in mitophagy; SIRT and PGC-1α are mutually influenced, while PGC-1α also regulates mitochondrial biogenesis.

At normal concentrations, ROS have an important role in cellular signaling; conversely, if ROS concentrations increase, as seen, for instance, in aging and in some aging-related diseases, they can cause DNA, protein, and lipid damage [43] that may lead to further mitochondrial dysfunction, followed by increased ROS generation and further damage, including changes in membrane permeability and membrane destruction, cytotoxic effects, and activation of DNA damage response, contributing to the process of aging [5,44]. Increased ROS levels observed in aging could be a result of age-related decline of mitochondrial functions [45]. Furthermore, high concentrations of ROS and insufficient cellular antioxidant defense cause oxidative stress that negatively affects the mitochondria in numerous ways [45,46], as shown in Figure 3. For instance, increased levels of ROS disturb the balance between mitochondrial fission and fusion, thus causing changes in mitochondrial morphology that have an effect on mitochondrial functions and may result in programmed cell death [45]. Furthermore, ROS reduces mitochondrial membrane potential, thus reducing the ATP production and activating apoptosis [45]. Increased levels of ROS activate mitogen-activated protein kinases and therefore induce senescence, which leads to aging progress [39]; additionally, ROS originating from damaged mitochondria can cause damage to telomeres that leads to compromised mitochondrial homeostasis and reduced expression of mitochondrial SIRT3-5 [47]. It has been suggested that longevity is correlated with mitochondria’s capacity to consume generated ROS: species that are not able to consume ROS have a shorter lifespan; as a result, lifespan could be extended via antioxidant supplementation [48]. There have been numerous studies where antioxidant molecules of plant origin reduced age-related syndromes [49]; the beneficial anti-aging effects of polyphenols exhibiting antioxidant properties are discussed in detail in the next section.

The implication of free radicals in the aging process represents one of the “classical” theories regarding aging [50], and while ROS have an important role in the aging process, newer theories suggest that there are multiple factors that can influence longevity [51].

### 3.2. Mitochondrial ETC and Aging

The activity, and stability, of respiratory chain complexes declines with aging [52], leading to increased ROS production [52]. More precisely, as mitochondrial function decreases, electron leakage from the ETC increases and produces higher levels of ROS [53]; simultaneously, ROS generation is less controlled in the aging process [40]. Some authors have suggested that mitochondrial oxidative damage represents one of the reasons for ETC dysfunction [54]. However, there is conflicting evidence that lifespan can be prolonged by both decreasing and increasing mitochondrial activity [55].

The activity of CI declines during the aging process due to the increased oxidation and reduced expression of complex subunits [42]. Actually, complex I is the most affected complex during the aging process, partially because most of its subunits are exposed to matrix ROS [40] and its suppression is corelated with reduced ATP synthesis and increased ROS production [45,56]. However, it seems that a decrease in complex I hydrophilic peripheral arm subunits leads to reduced superoxide synthesis and lifespan extension [42]. Depending on the studied tissue, it was noticed that complex I loses its activity in the brain, liver, and skeletal muscles, while complexes III and IV are altered in the heart muscle [52]. Another study conducted on skeletal muscles in orthopedic patients showed that, with aging, CI, CII, and complex I-dependent oxygen consumption decreases [42]. Interestingly, CI-IV can assemble and form “super-complexes”, thus reducing ROS production by CI [42].

As shown by multiple studies, the activity of CII declines with age; dysfunctional CII can also contribute to oxidative stress generation, as well as impaired energy production [57]. Studies have revealed that mutations of complex subunits that are encoded by nuclear DNA, such as succinate dehydrogenase complex subunit C (SDHC) and succinate dehydrogenase complex subunit B (SDHB), can lead to accelerated aging [58]. It is proposed that increased levels of ROS can cause nuclear DNA damage and, furthermore, affect subunit expression [58].

CIII deficiency has been shown to cause juvenile-onset progeroid syndromes that are characterized by decreased bone mineral density, kyphosis, alopecia, and thin skin [59]. Additionally, it was reported that some long-lived species present a lower level of ROS production, presumably due to a polymorphism in the cytochrome b sequence [60]. Actually, the production of ROS by CIII can be caused by age-related decline in CIV activity: slow-rate electron circulation can lead to an increased CoQ half-life, electron leakage, and ROS generation [40].

The relationship between ETC activity and other mitochondrial processes is represented in Figure 3.

### 3.3. Mitochondrial DNA and Aging

One of the causes of mitochondrial dysfunction in aging is represented by the accumulation of mitochondrial DNA (mtDNA) mutations [38] over a certain threshold where it influences lifespan and aging, causing physiological decline and pathological modifications [46]. Furthermore, mtDNA mutations are associated with ROS production, and both phenomena are more frequent with the aging process [3]. Compared to nuclear DNA, mtDNA is more prone to oxidative damage and mutations, firstly, because of its close proximity to ROS origins, and secondly, due to a deficiency in mtDNA repair systems [51,61]. ROS interacts with mtDNA (Figure 3) and can lead to the formation of DNA–protein crosslinks, base modification, and generation of single-strand breaks or double-strand breaks in the DNA [45]. The most common mutations caused by oxidative stress are conversions of guanine–cytosine to thymine–adenine and transitions of guanine–cytosine to adenine–thymine, with the former being observed as a prevalent mutation in aged mice [12]. Intriguingly, studies on Drosophila and aged human brains did not find that oxidative stress contributes to mtDNA mutations [12]. Collectively, mtDNA mutations can affect the stability or activity of respiratory chain CI, CIII, and CIV and even damage CV, thus reducing the synthesis of ATP. As a consequence, energy levels decline and cellular functions are affected, while protein synthesis and cellular repair mechanisms are altered, ultimately quickening the aging process. Furthermore, mitochondrial energy deficit leads to mobility impairment and physical frailty, some of the major challenges in aging [46]. For instance, the link between mtDNA mutations and aging was observed in an mtDNA mutator mouse that exhibited an increased number of mtDNA mutations and developed signs of premature aging like hearing loss, hair greying, hair loss, osteoporosis, and reduced lifespan [3]. Similarly, studies showed that the number of mtDNA copies declines with aging, with the copy number being an indicator of mitochondrial dysfunction and oxidative stress response [61]. This age-corelated decrease in the number of mtDNA copies was observed in both laboratory animals and in human skeletal muscles [62].

### 3.4. Mitochondrial Dynamics and Aging

Mitochondrial dynamics is a term that includes a number of mitochondrial processes, namely biogenesis, fission, fusion, transport, and mitophagy [63]. It was proposed that mitochondrial health can be preserved by activating mitophagy, fusion, and fission [19]. Mitochondrial biogenesis represents one of the major factors that regulates mitochondrial homeostasis, and its dysfunction can contribute to cellular aging. For example, impaired mitochondrial biogenesis could be the cause of reduced mtDNA copy number in the muscles of aged horses (Figure 3) [62]. Furthermore, the expression of PGC-1α, a factor that regulates mitochondrial biogenesis, was decreased in aged mice and horses [62].

Mitochondrial fission changes with the aging process; nevertheless, this change differs depending on the studied organism [64]. For example, in Saccharomyces cerevisiae, longevity was increased after the inhibition of fission [65]. Conversely, in *C. elegans* mice and human endothelial cells, it was reported that proteins that regulate fission decline with age; as a result, lifespan could be prolonged by re-establishing the levels of such proteins [64]. Mitochondrial fusion could delay aging by improving mitochondrial function [37]; thus, in *D. melanogaster* and *C. elegans*, increased fusion leads to extended longevity [65], but in Drosophila, the effects on longevity are not consistent [64]. Additionally, mitochondrial fission and fusion are corelated with other aging factors, such as cellular senescence, dysregulation of nutrient sensing, stem cell exhaustion, and genomic instability [66].

In the aging process, mitochondrial dynamics are being altered, and consequently, mitophagy is being inhibited; this results in the impaired clearance of dysfunctional mitochondria, their accumulation, and further mitochondrial damage, with negative effects on cellular functions [4]. More precisely, as levels of ROS increase with aging, mitophagy is activated, and damaged mitochondria are eliminated (Figure 3). Nevertheless, the quantity of damaged mitochondria can surpass the mitophagy capacity; as a result, these mitochondria produce excessive ROS that further accelerate the aging process [67]. Furthermore, the elimination of damaged mitochondria is followed by mitochondrial biogenesis from exclusively healthy mitochondria [68].

### 3.5. Mitochondrial Morphology and Aging

Mitochondrial morphology is subject to changes during the aging process, as several studies have showed—both enlarged mitochondria and fragmented mitochondrial network have been reported in aged hearts [63]. Similarly, in skeletal muscles of aged rats, aging was associated with increased mitochondrial branching, size and elongation (glycolytic tissue) and increased fission (in oxidative tissue) [69]. As mitochondrial function is linked to mitochondrial morphology, the enlargement of mitochondria and their branching leads to function impairment, like reduced ATP synthesis (Figure 3). Furthermore, mitochondrial elongation could be a possible explanation for mitochondria’s increased vulnerability to stress. Some of the reasons behind elongation could be an increase in fusion as a result of stress and lower bioenergetic efficiency or mitophagy impairment [70].

### 3.6. Mitochondrial Proteostasis and Aging

Proteostasis is characterized by protein quality control mechanisms and degradation pathways that are responsible for normal protein expression, folding, and turnover, which ultimately influence mitochondrial function. As proteostasis declines with age, ref. [71] mitochondrial proteostasis defense pathways become defected, leading to damage of the mitochondrial proteome. For example, as noticed in aged organisms, dysfunctional oxidative phosphorylation proteins can disturb both mitochondrial function and structure [72]. If proteins are terminally damaged, they undergo degradation; if proteins are misfolded or misassembled, protein folding chaperones can, in some cases, reverse the damage. One defense mechanism against proteotoxic stress is mitochondrial unfolded protein response (mtUPR) [72], which stands as a transcriptional response activated in the case of mitochondrial dysfunction with the aim of repairing the mitochondrial network [73]. Some of the triggers able to activate mtUPR include increased ROS (Figure 3), ETC dysfunction, mtDNA deletions, and accumulation of unfolded proteins [74]; its activation also leads to the initiation of mitochondrial proteostasis mechanisms, such as the production of antioxidants, proteases, and chaperones [75]. During aging, the activity of mtUPR declines, and thus, its activation could ameliorate age-dependent cell dysfunction [73]. Interestingly, mitochondrial stresses that activate mtUPT could have a prolonging effect on lifespan, as was shown in Drosophila, *C. elegans*, and mice [72].

### 3.7. Mitochondrial Permeability Transition Pore (mPTP)

Mitochondrial mPTP is a channel located in the IMM that can be activated by membrane depolarization, oxidative stress, and calcium ions that characterize age-related mitochondrial dysfunction; as a result, mPTP activity increases in aging. The activation of mPTP leads to the inhibition of oxidative phosphorylation due to proton-motive force collapse: protons enter the mitochondrial matrix, while hydrogen peroxide, superoxide, calcium ions, and respiratory substrates exit the matrix. Furthermore, large solutes flow into the matrix, causing its swelling and outer membrane rupture [76]. As a result of ROS, nicotinamide adenine nucleotide (NAD^+^), and calcium outflow, the level of oxidative stress in the cell rises, damaging DNA and proteins, subsequently leading to accelerated aging [2]. Recent studies have shown that mPTP can be inhibited by mtUPR activation [2].

### 3.8. Mitochondrial Membrane Potential (MMP)

MMP represents the electrochemical gradient between the inner and outer mitochondrial membrane; it is an important indicator of mitochondrial function, as its deregulation causes mitochondrial dysfunction [77]. Mitochondrial membrane potential declines during the aging process; moreover, it is associated with increased mitophagy and fission (Figure 3) [78]. If the mitochondrial membrane potential cannot be restored, depolarized mitochondria will be eliminated by mitophagy, and if a large number of mitochondria are affected, cell death will occur. As previously mentioned, the mitochondrial membrane depolarizes as a result of increased mPTP activation [2].

### 3.9. Mitochondrial Adenine Nucleotide Translocase (ANT) and Aging

ANT is a mitochondrial membrane ADP transporter that, along with mitochondrial ADP sensitivity, undergoes modifications with aging [41]. Considering that ROS production is correlated with ATP synthesis, which, in turn, depends on ADP availability, absence of ADP contributes to higher ROS levels. As previously demonstrated, the decline in ANT can be caused by ROS oxidation or hyperacetylation, leading to increased ROS production (Figure 3) and activation of the NF-κB pathway, negatively influencing mitophagy [41].

### 3.10. NAD^+^ and Aging

NAD^+^ is an enzymatic substrate and cofactor formed in the mitochondria during the Krebs cycle, glycolysis, and NADH oxidation by CI. NAD^+^ is essential for the proper function of a number of enzymes, including sirtuins. Research has indicated that its levels drop in the process of aging, and this decline impairs sirtuin activity [43,79]. Also, it was suggested that NAD^+^ decreases during aging as a consequence of chronic inflammation and increased oxidative stress [80]. NAD^+^ reduction causes the depolarization of the mitochondrial membrane and mitochondrial permeability transition [52] (Figure 3). Moreover, it increases the NADH/NAD^+^ ratio, resulting in reduced oxidative metabolism, higher ROS production, decreased mitochondrial biogenesis, and overall mitochondrial dysfunction. In this regard, numerous studies have shown that the restauration of NAD^+^ levels in mice leads to increased longevity [80].

### 3.11. Mitochondrial Regulators

#### 3.11.1. Sirtuins and Aging

Sirtuins represent a family of seven NAD^+^ dependent protein deacylases (SIRT1-7) that can be found in the mitochondria, nucleus, and cytosol [52,79]. Researchers have suggested a connection between the impairment of sirtuin function and the aging process, considering that sirtuins play an important role in antioxidative defence and DNA repair [43,81]. Mitochondrial sirtuins, SIRT 3-5, have a role in mitochondrial biogenesis, metabolism, and signaling [52,79]. The relationship between SIRT proteins and mitochondrial functions is represented in Figure 3.

SIRT 3 is localized mainly in the mitochondrial matrix but can also be found in the nucleus [82]; it regulates the electron transport chain and therefore influences ATP levels and also participates in acetyl-CoA formation [82]. Additionally, it was suggested that increased SIRT3 expression leads to the decline of ROS production and promotion of mitochondrial respiration [13]. More precisely, SIRT3 can activate superoxide dismutase (SOD2), which is able to transform superoxide to hydrogen peroxide; hydrogen peroxide is subsequently neutralized by glutathione, whose synthesis is also influenced by SIRT3 [43]. By increasing SOD2 activity, SIRT3 opposes aging [83]. Furthermore, this mitochondrial sirtuin activates fork-head box protein O 3A (FOXO3A) and thus induces the antioxidant program [43]. This effect was reported in an in vitro study on human umbilical vein and bovine aortic endothelial cells, wherein SIRT3 deacetylated FOXO3, protecting the mitochondria from oxidative stress, thus exhibiting potential age-delaying activity [84]. In addition, SIRT 3 deacetylates all ETC complexes and therefore reduces the production of ROS due to the defective electron transport [43]. Lastly, SIRT3 also has a role in mitochondrial biogenesis through the interaction with PGC-1α [19]. Due to its numerous effects, SIRT3 modulators are emerging as potential therapeutic options, research demonstrating that several polyphenols actively exhibit this activity [83].

SIRT4, another mitochondrial sirtuin, influences mitochondrial function in a number of ways [85]; however, when it comes to its effects on aging, results are somewhat contradictory. SIRT4 can be overexpressed in some aged tissues, such as oocytes, and downregulated in others, such as aged liver and brain [85]. Furthermore, its overexpression can lead to increased ROS and subsequent mitochondrial damage, but it can also decrease ROS production [85].

The last mitochondrial sirtuin, SIRT5, influences ROS levels by isocitrate dehydrogenase 2 (IDH2) desuccinylation and FOXO3A deacetylation [43]. A study on mice lacking SIRT5 showed reduced ATP production, while SIRT5 overexpression led to the restoration of ATP levels and improved mitochondrial function [86].

Although it is localized in the nucleus, SIRT1 has the ability to regulate mitochondrial homeostasis, promote mitochondrial function and restore disfunction, influence the production of ROS, and inhibit oxidative stress [86,87]. During the aging process, SIRT1 decreases together with mitochondrial biogenesis [88]. In addition, supporting this statement, studies have shown that SIRT1 overexpression prolongs cellular lifespan [87].

#### 3.11.2. FOXO and Aging

FOXO are a family of four proteins, FOXO1, FOXO3, FOXO4, and FOXO6, with FOXO1 and FOXO3A localized in the mitochondria, where they can bind to mtDNA [89]. The FOXO family regulates mitochondrial function by influencing mitochondrial biogenesis, mitochondrial fusion and fission, and mitophagy [90]; therefore, FOXOs also play a role in aging. For example, the FOXO3 gene locus is associated with centenarians, with FOXO1 also being correlated with human longevity [91]. Furthermore, it was shown that FOXO1, FOXO3, and FOXO4 deletions lead to increased ROS production [92]. In addition, FOXO-induced mitophagy could represent a helpful factor during aging [91]. The influence of FOXO on other mitochondrial processes is represented in Figure 3.

#### 3.11.3. PINK1 and Aging

PINK1, or (PTEN)-induced putative kinase protein 1, represents a mitochondrial kinase with a role in mitochondrial quality control that protects against mitochondrial dysfunction [93]. In fact, the PINK1/Parkin pathway is one of the most important mitophagy pathways, and its activation could have a role in the treatment of age-associated impairments, considering that PINK1/Parkin expression decreases in Alzheimer’s disease and osteoarthritis while also playing a role in Parkinson’s disease [94]. By activating this pathway, age-related decline in mitophagy can be restored [94].

#### 3.11.4. AMPK and Aging

AMPK or AMP-activated protein kinase is a complex activated when ATP decreases, ultimately leading to an increase in ATP synthesis [95]. It represents a point of interest in anti-aging research owing to its ability to delay or stop the aging process when activated, as was recorded in most model organisms. In addition to its role in energy metabolism, AMPK can promote mitophagy; moreover, the AMPK pathway can activate both FOXO proteins and SIRT1 (Figure 3) [96].

## 4. Targeting Mitochondria with Polyphenols to Mitigate Aging-Related Decline

**Figure 4:**
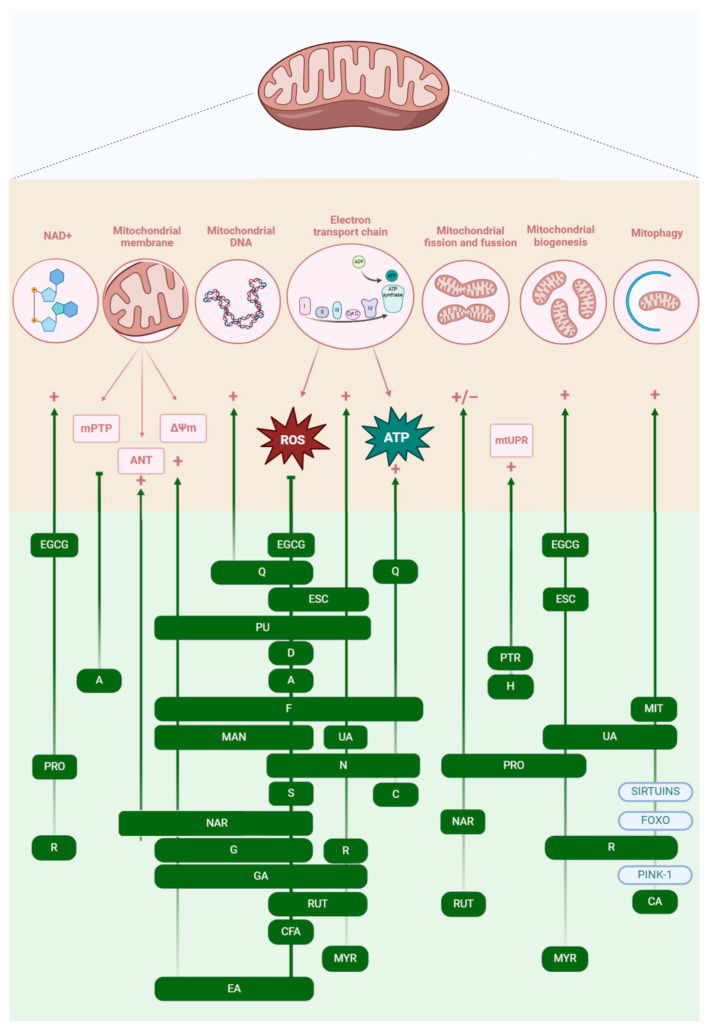
Different mechanisms of polyphenols affecting mitochondrial function in aging. EGCG—epigallocatechin 3-gallate; Q—quercetin; ESC—esculetin; PU—punicalagin; D—delphinidin; A—apigenin; F—fisetin; H—hesperetin; PTR—pterostilbene; MAN—mangiferin; N—nobiletin; S—sesamin; C—curcumin; UA—urolithin A; NAR—naringenin; MYR—myricetin; CA—catechinic acid; CFA—caffeic acid; EA—ellagic acid; GA—gallic acid; G—genistein; R—resveratrol; RUT—rutin; MIT—mitophagy-inducing coumarin; and PRO—procyanidins.

The protective role of polyphenols in the aging process originates from the relationship between ROS and aging, as polyphenols act as antioxidants that could scavenge ROS and thus slow down the aging process [20]. Polyphenols have antioxidant properties due to the presence of their phenolic groups [97]; although the antioxidant activity varies based on the number and position of the hydroxyl group [37], they can directly eliminate ROS or increase the expression of catalase and superoxide dismutase [32]. However, studies have shown that some polyphenol compounds also have the ability to influence other mitochondrial processes, such as mitophagy, mitochondrial biogenesis, and mitochondrial fusion and fission (Figure 4) [32].

### 4.1. Phenolic Acids

#### 4.1.1. Caffeic Acid

Caffeic acid, found in tea, coffee, and wine, has been reported by multiple studies as antioxidant agent [98]; the link between its antioxidant ability and aging was suggested by Saenno et al., who tested the effects of caffeic acid on D-galactose-induced aging in rats and reported improved memory deficits and neural apoptosis. Such effects were attributed to the decrease in the activity of malondialdehyde and increase in that of antioxidant enzymes such as SOD and glutathione peroxidase [98]. Another study reported that caffeic acid increases catalase and SOD activity in aged *Drosophila*, delaying the age-associated modifications in intestinal stem cells [99].

#### 4.1.2. Gallic Acid

Gallic acid can be found in berries, grapes, and wine [100]. When tested on a WS hMSC accelerated aging model, gallic acid decreased mitochondrial ROS levels and increased MMP. It also reduced mitochondrial mass [101]. Another study also showed that gallic acid has an ability to reduce oxidative stress by increasing the levels of total thiol molecules and enhancing the activity of mitochondrial CI, II, and IV in H_2_O_2_-induced aging [100].

### 4.2. Flavonoids

#### 4.2.1. Apigenin

Apigenin is mostly found in onions, oranges, and wheat; Oyebode et al. [102] studied the protective role of apigenin on D-galactose-induced aging in *Drosophila melanogaster*. The results confirmed that apigenin delays the aging process due to its antioxidant activity, reduces the amount of accumulated hydrogen peroxide, and also restores the glutathione-S transferase and catalase activities. Moreover, apigenin can inhibit the opening of the mPTP caused by D-galactose administration [102], thus revealing that the anti-aging effect of apigenin can be attributed to its mechanism at the mitochondrial level.

#### 4.2.2. Nobiletin

Nobiletin is a flavonoid originating from the peels of citrus fruits [103]. Nohara et al. tested the anti-aging effects of nobiletin in aged mice; the results showed that the mice fed with nobiletin and on a high fat diet exhibited improvement of aging-related markers like exercise endurance, energy expenditure, and grip strength. Furthermore, nobiletin improved mitochondrial respiratory complex function in skeletal muscles, restored super-complex formation, depending on high-fat-diet consumption, and induced the expression of genes encoding components of the mitochondrial respiratory complex. By activating mitochondrial respiration, nobiletin has a role in decelerating metabolic aging [104]. Another study that confirms nobiletin’s effects was performed by Wang et al. [103]; by inducing oxidative aging in C2C12 myoblasts with D-galactose, nobiletin reduced ROS generation and improved mitochondrial function by restoring the normal levels of ATP production and basal and maximum respiration, thus presenting antioxidant properties that ultimately lead to a delay in D-galactose-induced aging [103].

#### 4.2.3. Genistein

Genistein is an isoflavone found in soybeans; due to its antioxidant properties, it is a promising anti-aging compound against skin aging. In one study conducted on keratinocytes and fibroblasts, it was found that genistein could prevent skin aging by reducing ROS release and preventing the fall of MMP [105].

#### 4.2.4. Quercetin

Quercetin belongs to the flavanol class of polyphenols, and it is naturally abundant in onions, apples, berries, and asparagus. Like many other polyphenols, it exerts antioxidant activities due to its chemical structure: the hydroxyl group has the ability to scavenge ROS, preventing later oxidation of molecules and thus exerting a protective effect against aging [106]. One study evaluated the aging effects of Bisphenol S on *C. elegans* and investigated quercetin’s role as a protective factor. Quercetin extended both a shortened lifespan, caused by Bisphenol S, and a shortened mean lifespan. Furthermore, quercetin reversed Bisphenol S-induced mitochondrial injury: it increased the mitochondrial content, as well as mtDNA concentration and ATP production, and decreased the mitochondrial fragmentation rate and ROS production. Quercetin also upregulated the expressions of IIS/daf-16 and antioxidative genes [107].

#### 4.2.5. Rutin

Rutin (quercetin-3-O-rutinoside, vitamin P) can be found in buckwheat seeds and citric fruits [37], and it exhibits antioxidant activities, as reported by Girsang et al. [108]. In agreement with this observation, rutin prolonged the lifespan of *Drosophila* and *C. elegans* by exhibiting antioxidant and anti-inflammatory effects [37]. Furthermore, when tested in aged rats, rutin inhibited oxidative stress by increasing superoxide dismutase and glutathione levels, and it also inhibited mitochondrial dysfunction by blocking the decrease in oxygen consumption [109]. Another effect on mitochondria was observed when sodium rutin positively influenced the expression of respiratory chain genes when assessed in mice in the context of aging [110]. Although not tested for its anti-aging effects, rutin did downregulate the expression of the DRP1 protein, therefore suppressing mitochondrial fission [37].

#### 4.2.6. Fisetin

Fisetin is a flavonoid synthesized in onions, apples, and strawberries; studies have shown it possesses the ability to prolong lifespan in *C. elegans* [111]. Indeed, fisetin reduced the level of ROS and susceptibility to oxidative stress [111]. Similarly, fisetin reduced ROS in aged oocytes by upregulating SIRT1. As a result, it restored MMP and the expression of respiratory chain genes, leading to increased ATP [112].

#### 4.2.7. Myricetin

Myricetin is a polyphenol from the flavanol group, present in tea and berries. A study conducted in mice and *C. elegans* showed that myricetin activates SIRT-1 and consequently deacetylates PGC-1α, leading to the following effects: in mice, myricetin improves endurance, while it elongates lifespan and healthspan in *C. elegans*. More precisely, it increases mitochondrial function through OXPHOS gene activation and mitochondrial biogenesis induction [113].

#### 4.2.8. Catechinic Acid

Catechinic acid is a polyphenol that can be found in tea, as well as fruits [114]. It activated mitophagy in *C. elegans* through BEC-1 and PINK-1 activation, thus prolonging lifespan and inhibiting age-associated behaviors [115].

#### 4.2.9. Epigallocatechin 3-Gallate

Epigallocatechin 3-gallate (EGCG) originates from green tea; one study showed it extended the lifespan of *C. elegans* in the following manner: EGCG initially increased ROS levels, which subsequently activated adaptive response via superoxide dismutase and catalase increase. This action led to a decline in ROS levels. Furthermore, EGCG-treated *C. elegans* showed an increase in mtDNA/nuclear DNA ratio and in the cts-1 gene, in relation to the mitochondrial biogenesis. EGCG increased the levels of NAD^+^ through the AMPK/AAK-2 pathway. However, it should be mentioned that FOXO/DAF-16 was required for Epigallocatechin 3-gallate lifespan extension [116]. Also, it was noticed that EGCG influences sirtuin- and cAMP/PKA-dependent mechanisms and thus stimulates oxidative phosphorylation. Moreover, it can promote mitochondrial biogenesis [117].

#### 4.2.10. Hesperetin

Hesperetin, the aglycone form of hesperidin, is a flavonoid found in many citrus fruits. In one study, hesperetin prolonged the lifespan of *C. elegans* previously exposed to chronic oxidative stress; in terms of the underlying mechanism, the researchers suggested that hesperetin activates mtUPR [118]. Another study showed that hesperetin prolongs lifespan in naturally aged mice through Cisd2 activation, thus maintaining mitochondrial function and integrity [119].

#### 4.2.11. Naringenin

Naringenin is a citrus-originating flavanone [120] whose chronic administration in mice significantly activates SIRT1 and reduces ROS levels, thus protecting the myocardium against age-associated dysfunction [120]. Moreover, naringenin has also shown effects against age-related retinal degeneration in 12-month-old mice; it improved retinal structure and visual function by decreasing mitochondrial fission, increasing mitochondrial fusion, and inhibiting mitochondrial damage [121] Wang et al. suggested that naringenin has the ability to improve ANT activity and increase MMP [122]; although the study itself was not corelated with anti-aging effects, the reported modified ANT activity and decreased MMP are both linked to the aging process [41,78].

#### 4.2.12. Delphinidin

Delphinidin can be found in pigmented fruits and vegetables [123]. Based on its antioxidative properties, Chen et al. investigated delphinidin’s effects on age-associated cardiac hypertrophy. The results showed that delphinidin reduces ROS in aged mice by decreasing the production of superoxide and NADPH oxidase activity [123].

### 4.3. Curcuminoids—Curcumin

Curcumin is a polyphenol originating from turmeric rhizome [124]. It was shown that curcumin enhances mitochondrial function in SAMP1 mice [19]. When tested on yeast, curcumin increased the levels of ATP, thus improving mitochondrial function and prolonging lifespan [124].

### 4.4. Coumarins

#### 4.4.1. Esculetin

Esculetin is a polyphenol belonging to the coumarin subclass, found in *Fraxinus spp* [125]. Similar to other polyphenols, esculetin showed anti-aging activity by inhibiting ROS in human keratinocytes [125]. In order to overcome its low bioavailability, Pulipaka et al. synthesized mitochondria-targeted esculetin, an esculetin-conjugated cation. When tested on human aortic endothelial cells treated with H_2_O_2_, conjugated esculetin activated the AMPK signaling pathway, leading to increased SIRT3 expression and improved mitochondrial biogenesis while simultaneously restoring inhibited mitochondrial respiration [126].

#### 4.4.2. Urolithin A

Urolithin A, a metabolite of ellagic acid, is produced under the influence of gut microbiota. When tested on *C. elegans*, a study showed that urolithin A preserved the respiratory capacity, stimulated mitophagy, and maintained mitochondrial biogenesis, thus prolonging lifespan [37]. Additionally, urolithin A promoted mitophagy in Mode-K intestinal cells and C2C12 myoblasts; furthermore, the same authors demonstrated that despite inhibiting CI, urolithin A enhanced CII respiration and upregulated the subunits of CII, III, IV, and V [127]. Through all these mitochondrial mechanisms, the authors revealed that urolithin A positively influenced muscle function in aged mice [127]. Urolithin A also showed its anti-aging properties when tested in elderly humans; after the administration of 500/1000 mg of urolithin A for 4 weeks, the mitochondrial gene expression and biogenesis were upregulated in the skeletal muscle, thus improving mitochondrial health [128].

#### 4.4.3. Mitophagy-Inducing Coumarin

Mitophagy-inducing coumarin (MIC) is a plant-derived, benzocoumarin compound able to prevent mitochondrial dysfunction and induce mitophagy in *C. elegans*, thus prolonging its lifespan [129]. In the same paper, the authors studied the possible mechanisms that lie beyond mitophagy activation; the results showed that dct-1/BNIP3 and daf-12/FXR were necessary for MIC-induced mitophagy. Furthermore, MIC increased the maximal respiratory capacity by eliminating damaged mitochondria [129].

### 4.5. Stilbenes

#### 4.5.1. Resveratrol

Resveratrol is a polyphenol from the stilbene group found in red wine, grapes, blueberries, bananas, cocoa, etc. Numerous studies have shown that resveratrol influences mitophagy through multiple mechanisms, depending on the tested species. For example, in rat heart tissue, resveratrol upregulated SIRT3 and FOXO3 [130]; in the H9c2 cardiac myoblast cells of piglets, rats, and zebrafish, resveratrol increased the quantity of PINK1 [130]. There is evidence suggesting that mitophagy upregulation is the underlying mechanism that contributes to the clearance of aged and dysfunctional mitochondria, with the potential to improve age-associated cognitive impairment [130]. Another study showed that grapes, containing resveratrol, trigger mitophagy through the Sirt1-Sirt3-Foxo3-PINK1-PARKIN pathway as a consequence of induced fusion and fission balance, therefore exerting anti-aging activity [131]. Furthermore, resveratrol also prevents oxidative stress-induced decline of mitophagy that develops during aging; this effect was possible due to its antioxidative activity [130]. Moreover, resveratrol activates SIRT1 and PGC1α, thus upregulating OXPHOS genes and increasing mitochondrial biogenesis. These modifications are expressed through increased running capacity in resveratrol-treated mice [132]. Similarly, it upregulated mitochondrial biogenesis in aged cow’s oocytes, improving their quality and potentially contributing to the prevention of maternal aging [24]. Furthermore, resveratrol prolongs lifespan by increasing AMPK and PGC1α in high-calory-fed mice [133]; it also increases NAD^+^ levels [134]. A phase 2 clinical study revealed that the daily intake of resveratrol improves mitochondrial function in skeletal muscle, as well as mobility during aging [32]. Nevertheless, resveratrol presents poor bioavailability if administered to humans; therefore, its clinical utility is rather limited. Besides clinical trials that have shown promising results, there are also conflicting cases [135]. For example, when tested in non-obese or healthy obese individuals, resveratrol did not show beneficial metabolic effects, suggesting that resveratrol exhibits its activity where there is a certain degree of metabolic abnormalities [136]. Another study, performed on 783 community-dwelling elderly people, proposes that dietary resveratrol does not show a substantial influence on longevity [137].

#### 4.5.2. Pterostilbene

Pterostilbene is a compound found in blueberries [138]; as previously reported, pterostilbene, specifically in combination with a mitochondrial cocktail, has the ability to activate mtUPR and increase sirtuin activity, leading to lifespan extension [37].

### 4.6. Lignans—Sesamin

Sesamin is a lignane found in sesame [139]; it exerts its anti-aging effects by inhibiting ROS accumulation. Namely, sesamin upregulated several antioxidative genes (catalase, Sod1 and Sod2), leading to anti-aging effects at the muscular and brain level in a senescence-accelerated *Drosophila* model [139]. Similar results were obtained by Shimoyoshi et al., who reported that senescence-accelerated mice fed with a combination of sesamin and episesamin showed an amelioration of age-related brain dysfunction that can be attributed to the antioxidant activity of lignans [140].

### 4.7. Tannins

#### 4.7.1. Punicalagin

Punicalagin is an ellagitannin originating from pomegranate [141] able to improve memory and learning deficits in aged mice by reducing ROS levels [142]. In addition, punicalagin maintained ETC activity, preserved the MMP, and reduced ROS production in retinal pigment epithelium cells previously treated with H_2_O_2_ [143].

#### 4.7.2. Ellagic Acid

Ellagic acid originates from raspberries, pomegranates, and walnuts [37]. It has been described as a highly efficient antioxidant able to scavenge O_2_^•^, OH^•^, and ROO^•^ radicals [144]. Hseu et al. showed that ellagic acid protects normal HaCaT keratinocytes against UV-induced oxidative stress in the following manner: due to its antioxidative properties, it increases HO-1 and SOD levels, reduces ROS, inhibits apoptosis, and, therefore, increases cell viability. Moreover, it prevented the reduction of MMP; as a result, authors have proposed ellagic acid as a potential treatment in UV-induced skin aging [145].

#### 4.7.3. Procyanidins

Procyanidin is a condensed tannin composed of catechin and epicatechin units; it can be found in a variety of fruits and vegetables, like apples, grapes, mangoes, soybeans, and barley [146]. In a senescence-accelerated mice model, procyanidins influenced mitochondrial fusion, fission, and biogenesis, thus improving mitochondrial quality [146]. Moreover, grape seed procyanidins improved mitochondrial function by increasing NAD^+^ content and thereby promoting SIRT1 expression in the aged mice [146].

### 4.8. Xantonoids—Mangiferin

Mangiferin is a polyphenol found in *Mangifera indica;* one study indicated that preconditioning human dermal fibroblasts with mangiferin protected the cells against H_2_O_2_-induced premature senescence: mangiferin decreased ROS levels, re-established the MMP, and maintained the mitochondrial mass. This protective effect could be attributed to mangiferin’s ability to scavenge free radicals [77].

### 4.9. Mixtures of Polyphenols

The anti-aging effects of various polyphenolic mixtures are presented in Table 2. 

## 5. Future Perspectives

Aging has become one of the most important concerns and points of interest for modern medicine [156]. Not only does the aging process cause many age-related diseases, but it also creates a burden for the health care system, especially as the number of elderly people will continue to rise in the future [156]. Although lifestyle interventions could contribute to healthy aging, lifestyle-independent interventions, such as therapeutics, could reverse the aging process and bring supplementary health benefits [156]. As we discussed in the present study, polyphenols are a vast group of compounds that have the ability to influence multiple age-associated targets, thus making them a viable anti-aging option. Additionally, with the scientific progress that is being made on a molecular level, new mechanisms of action in aging are being discovered, and new drugs that can modulate those mechanisms will need further study. Hence, polyphenols represent a major source of inspiration for scientists due to their versatile activity and large number of compounds, many of which have not been tested yet in the anti-aging context. We focused on the effects of polyphenols on mitochondrial function, as mitochondria have numerous roles that are crucial for the normal functioning of the organism, as well as the aging process. Polyphenols have been shown to target mitochondrial dysfunction both in vitro and in vivo, exerting effects on mitochondrial ROS, ATP levels, ETC, mtDNA, MMP, mPTP, NAD+, mitochondrial biogenesis, fusion, fission, and mitophagy. Furthermore, in one study, they ameliorated some of the age-induced alterations, such as memory deficit, visual function, and exercise endurance, and ultimately, their use led to life extension. The most studied and most prevalent effect shown by polyphenols was the antioxidant effect, which is attributed to the phenolic hydroxyl group [157]. More precisely, the OH group donates its H+ to the free radicals, neutralizing them [158]. The respective stabilization of radicals enables their clearance by cellular antioxidants, like glutathione [159]. This, as well as other antioxidant activities attributed to polyphenols (metal chelation, inhibition of oxidant and induction of antioxidant enzymes), leads to ROS removal or inhibition of ROS formation [158]. The antioxidant activity can be influenced by the position and number of hydroxyl groups [160], and at the same time, masking of critical hydroxyl groups can lead to the absence of antioxidant effects [161]. This notion was confirmed in a study carried out by Santos et al., where the antioxidant activity of 18 examined polyphenols increased as the number of polyphenol groups rose [160]. Moreover, it seems that lipophilicity and polarization intensify antioxidant properties, while polyphenols’ ability to donate electrons [160], their molecular properties, and their steric effects [160] also contribute to antioxidant capability. For example, stilbenoid derivatives that present an electron-donating group in their structure, like 3-methoxy, have shown higher antioxidant activity [159]. Furthermore, flavonoids that are characterized by the presence of a C^2^=C^3^ double bond and a 3-OH group have the ability to donate electrons more efficiently and exerted a stronger antioxidant activity compared to compounds that lacked these moieties [159]. Moreover, it was suggested that the presence of trans-alkene functionality is required for resveratrol’s potent antioxidant properties [159]. The position of the hydroxyl groups also influences polyphenols’ activity: some flavonoid and stilbenoid compounds that contain an ortho-dihydroxyl group have better antioxidant capabilities due to hydrogen bonding and radical stabilization compared to compounds with two non-neighboring hydroxyl groups [159]. In addition, hydroxyl groups, as well as phenyl rings, interact with amino acid residues of SIRT1, as was observed in the case of resveratrol [161]. In other molecules, such as quercetine, the selectivity and affinity for Sirt6 could be improved by C-ring modifications [162]. Therefore, adequate structural modifications could lead to an increase in antioxidant activity and possibly strengthen the anti-aging effect. As we have discussed in this paper, ROS plays an important role in the aging process and also influences other mitochondrial processes; hence, by decreasing oxidative stress, polyphenols could ameliorate aging through multiple interconnected mechanisms. As was observed in the previously mentioned studies, by enhancing mitochondrial functions, polyphenols were able to improve age-associated tissue and organ dysfunctions. Moreover, polyphenols reduced UV-induced oxidative stress and presented effects in aged models, indicating that they can positively influence both photoaging and chronological aging. Nevertheless, more detailed studies are needed to elucidate how exactly polyphenols are able to influence mitochondrial function, with the structure–activity relationship requiring special attention. To further improve the effects of polyphenols on mitochondria, new mitochondria-targeting compounds that can selectively accumulate in the mitochondria can be synthesized. This can be achieved by conjugating polyphenols with triphenyl phosphonium (TPP+), a membrane-permeable cation. There have been several studies wherein polyphenols such as resveratrol, quercetin, and caffeic acid have been conjugated with TPP+, leading to improved mitochondrial activity, with resveratrol having an increased mitochondrial uptake up to 500 folds [163]. Moreover, new nanotechnologies could be used in order to enhance the delivery of polyphenols to mitochondria. Some types of mitochondria-targeted nanocarriers have already been used to incorporate polyphenols and have been tested in disease-associated mitochondrial dysfunction from cancer and myocardial ischemia–reperfusion injury. Such examples include solid lipid nanoparticles containing ellagic acid, as well as polymer nanosystems with resveratrol and curcumin [164]. Anti-aging treatments could benefit from the use of these formulations, as they have also been tested on non-cancerous cell lines and could provide more potent effects on mitochondria with the use of smaller concentrations. Although polyphenols showed promising effects on cell lines and animal models, there is an insufficient number of clinical studies. Aspects such as possible adverse reactions, dosage, and treatment duration need to be further examined. The clinical use of polyphenols is limited due to certain challenges, such as low bioavailability [97], rapid metabolization, or poor solubility [165]. However, there are methods, such as structural modifications, that can improve the bioavailability. Some of the possible alterations of the molecule include alkylation, carboxymethylation, acetylation [97], glycosylation, conjugation with lipids and proteins, or the use of different nanocarriers [165], all of which can influence the bioavailability and, therefore, polyphenols’ therapeutic efficiency [166].

Future directions in this field include the biosynthesis and development of polyphenol derivatives with improved bioavailability and anti-aging effects; these efforts should be guided by structure–activity relationship studies and include modern technologies, such as nanotechnology-based delivery systems. Additionally, given the essential role of mitochondria in aging, it is imperative to assess the effects of these novel compounds on mitochondrial function and their role in alleviating age-related dysfunction.

## 6. Conclusions

Polyphenols represent a significant group of natural compounds that have shown promising anti-aging effects due to their ability to modulate numerous aging hallmarks. Multiple polyphenolic classes—phenolic acids, flavonoids, coumarins, curcuminoids, stilbenes, lignans, tannins, and xanthonoids—can cause an improvement of age-related mitochondrial dysfunction, both in vitro and in vivo. More precisely, they can reduce ROS levels; improve ATP synthesis and ANT activity; modulate ETC function, fission, and fusion; restore MMP; promote mitophagy and mitochondrial biogenesis; increase NAD^+^ levels; inhibit mPTP; and activate mtUPR. The most prevalent effect is ROS targeting, associated with polyphenols’ antioxidant activity, which, in turn, is attributed to the phenolic group present in the molecule. Although polyphenols exhibit an anti-aging effect through mitochondrial dysfunction targeting, more studies are needed to further investigate new polyphenolic compounds, possible mechanisms of action, and effects in humans, while also addressing certain challenges, like low bioavailability.

## Figures and Tables

**Table 1:** Effects of polyphenols on different aging hallmarks.

Polyphenolic Compound(s)	Sample Type	Mechanism of Action	Anti-Aging Effects	Reference
**Epigenetic alterations**				
Genistein	Mouse fibrotic kidneys	↓ histone 3 deacetylation of Klotho ↓ DNMT1, DNMT3a	↓ renal fibrosis, a typical renal aging feature	[27]
Rosmarinic acid	Human Skin Fibroblasts	↑ 5-mC in late passage cells	Aging-modulatory effects	[28]
**Telomere attrition**				
Resveratrol, red wine	Male Wistar rats	↑ telomere length and activity	Delayed vascular aging, no lifespan effect	[29]
Resveratrol	Human aortic smooth muscle cells	Telomerase activation via NAMPT-SIRT4	Potential anti-aging effect on aortic smooth muscle cells	[30]
Rosmarinic acid	Serially passaged Human Skin Fibroblasts	↓ rate of telomeres loss	Aging-modulatory effects	[28]
Curcumin	Mesenchymal stem cells derived from adipose tissue	↑ telomerase reverse transcriptase expression	Improved lifespan of cells	[31]
**Stem cell exhaustion**				
Oleuropein	Human bone marrow mesenchymal stem cell progenitors	↑ osteoblastogenesis ↓ adipogenesis	Slowing skeletal aging	[32]
Curcumin	Mesenchymal stem cells derived from adipose tissue	Proliferation of stem cells	↓ cell aging	[31]
**Cellular senescence**				
Oleuropein	Human lung cells Neonatal human dermal fibroblasts	↓ number of prosenescent cells	Potential beneficial effects on aging	[33]
Oleuropein	Human embryonic fibroblast IMR90	Delayed senescence morphology development	Prolonged lifespan	[32]
Genistein	Human umbilical vein endothelial cells	Inhibited senescence ↓ p16, p21, SA-β-gal	Potential aging-delaying effects	[34]
**Altered intercellular communication**				
Resveratrol	Aged mice	↓ TNF-α and IL-1ß	↓ of age-related proinflammatory pattern	[32]
Epigalocathecin	Male Swiss albino mice	↓ TNF-α and IL-1ß	Enhanced lifespan and healthspan	[35]
**Deregulated nutrient sensing**				
Catechins	Aged rats’ hippocampus	↑ SIRT 1	Improved cognitive abilities	[32]
Epigalocathecin	Male Swiss albino mice	↓AMPK, AKT ↑ SIRT 3,5	Enhanced lifespan and healthspan	[35]
**Loss of proteostasis**				
Resveratrol	CuSO_4_-SIPS WI-38 fibroblasts	Improved proteostasis	Possible prevention of age-associated cellular dysfunction	[36]
**Dysbiosis**				
Epigalocathecin	Male Swiss albino mice	Preserved microbial diversity ↓ pathogenic/opportunistic pathogenic species	Enhanced lifespan and healthspan	[35]
Quercetin	Mice with pulmonary fibrosis	Improved intestinal flora imbalance	Delayed the aging process of alveolar epithelial cells	[37]
**Chronic inflammation**				
Pomegranate	Aged mice	↓ IL-6, IL-1β, IL-18, TNF-α,	Improved memory and learning deficits	[37]

**Table 2:** The anti-aging effects of various polyphenolic mixtures.

Polyphenolic Compounds	Origin	Sample Type	Mechanism of Action	Anti-Aging Effects	Ref.
Oleuropein, oleurosid, and hydroxytyrosol	*Olive oil*	NMRI mice	Increased the age-declined level of ATP in brain cells	Improved spatial working memory	[147]
Oenothein B and pentagalloyl glucose	*Eucalyptus polyphenols*	*C. elegans*	Regulated the ETC by influencing the ETC encoding gene, isp-1	Extended the lifespan	[26,148,149]
Catechin, epicatechin, proanthocyanidins, and trans-resveratrol	Grape skin extract	C57BL/6J mice aged brain	Increased moderately the ATP levels Partly improved mitochondrial respiration	Shift in survival curve toward higher survival rates	[150]
Anthocyanins	*Aronia melanocarpa*	Aged mice	Increased the level of SOD and glutathione peroxidase (GPx)	Inhibited age-related cognitive decline	[151]
Myricetin, quercetin, kaempferol, and isorhamnerin glycosides	*Nelumbo nucifera*’s stamen extract	*Saccharomyces cerevisiae*	Upregulates SIRT and SOD → reduced oxidative stress Preserved MMP, contributing to the maintenance of mitochondrial functions	Delayed chronological aging	[152]
Hydroxicinammic acid derivates (5-O-caffeoylquinic and 5-O-feruloylquinic acids)	Green coffee extract	*C.elegans*.	Prevented oxidative stress	Delayed aging Lifespan prolongation	[153]
21 compounds (protocatechuic acid)	Pre-fermented polyphenol mixture	Mice, *C.elegans*	Increased the activity of CI, II, and IV Increased MMP and ATP concentration	Increased the median lifespan in both species	[154]
Rutin, ellagic acid, kaempherol, cyanidin, malvidin, and delphinidin	Fruit/berry/vegetable juice powder		Reduced ROS Increased SOD, catalase, GPx, and heme oxygenase-1 levels	Attenuates aging	[155]

## Data Availability

No new data were created or analyzed in this study.

## Abbreviations

The following abbreviations are used in this manuscript:

AMP	Adenosine monophosphate
AMPK	AMP-activated protein kinase
ANT	Adenine nucleotide translocase
ATP	Adenosine triphosphate
BEC-1	Beclin-1
CI	Complex I
CII	Complex II
CIII	Complex III
CIV	Complex IV
CV	Complex V (ATP-synthase)
EGCG	Epigallocatechin 3-gallate
ETC	Electron transport chain
FMN	Flavin mononucleotide
IMM	Inner mitochondrial membrane
MIC	Mitophagy-inducing coumarin
mtDNA	Mitochondrial DNA
MMP	Mitochondrial membrane potential
mPTP	Mitochondrial permeability transition pore
mtUPR	Mitochondrial unfolded protein response
OMM	Outer mitochondrial membrane
PGC-1α	Peroxisome proliferator-activated receptor gamma coactivator 1-alpha
PTEN	Phosphatase and tensin homolog
ROS	Reactive oxygen species
SIRT	Sirtuin
SOD	Superoxide dismutase
SDHB	Succinate dehydrogenase complex subunit B
SDHC	Succinate dehydrogenase complex subunit C
TPP+	Triphenyl phosphonium
